# Integrative Discussion: Clinical Importance and Applied Potential of including Diversity in Resilience Research

**DOI:** 10.1002/alz70860_099975

**Published:** 2025-12-23

**Authors:** Prashanthi Vemuri

**Affiliations:** ^1^ Department of Radiology, Mayo Clinic, Rochester, MN, USA

## Abstract

**Background:**

The first three talks focused on three important aspects on including diversity in Resilience Research ‐ engagement and inclusion of historically underserved communities, including sex, gender, and social determinants of health (SDH) in our analyses, and methodological challenges and recommendations for inclusion of diverse populations.

**Objective:**

While important knowledge gaps were identified and recommendations made for diversity inclusion, discussion of these topics in the context of clinical importance and applied potential will enhance knowledge mobilization and translation of research findings which will be discussed as part of this talk.

**Methods:**

Clinical importance for knowledge mobilization focuses on direct impact on patient diagnosis/treatment to ensure research improves health outcomes. Whereas, applied potential focuses on public health and outreach activities to ensure that research reaches underserved communities and subgroups of populations.

**Results:**

Guided by the aspects discussed in the first three talks, we will discuss specific examples from emerging research across diverse populations on 1) how inclusion of diversity in resilience research can aid in improvements in patient care, screening, and management; and 2) how better data collection and targeting awareness of research in underserved communities along with improving instruments of recruitment and retention can provide us with data for deeper evaluation of mechanisms of prevention across different sub‐populations.

**Conclusion:**

While creating a roadmap for inclusion of diversity in resilience research for aging and dementia is the important first step, focus should be placed on effective translation of the emerging research findings to reach broader aspects of patient care.

